# Biochemical pathway analysis of gastric atrophy 

**Published:** 2018

**Authors:** Mostafa Rezaei Tavirani, Sina Rezaei Tavirani, Fatemeh Tajik Rostami

**Affiliations:** 1 *Proteomics Research Center,Faculty of Paramedical Sciences, Shahid Beheshti University of Medical Sciences, Tehran, Iran*; 2 *Faculty of Medicine, Iran University of Medical Sciences, Tehran, Iran *

**Keywords:** Gastric atrophy, Network analysis, Gene, Biochemical pathway, Central node.

## Abstract

**Aim:**

Pathway analysis of gastric atrophy to find new molecular prospective of disease.

**Background:**

Gastric atrophy as a process which is accompanied with “loss of glans” in stomach can be considered as a risk factor of gastric cancer. Here, the correlated biochemical pathways to the disorder have been analyzed via protein-protein interaction (PPI) network analysis.

**Methods:**

The genes related to gastric atrophy were retrieved by STRING database and organized in a network by Cytoscape. Three significant clusters were determined by ClusterONE plug-in of Cytoscape. The elements of cluster-2 which contained all central nodes of the network were enriched by ClueGO and the biochemical pathways discussed in details.

**Results:**

The number of seven central nodes (which are included in cluster-2); INS, TP53, IL6, TNF, SRC, MYC, and IL8 were identified. The biochemical pathways related to the elements of cluster-2 were determined and clustered in nine groups. The pathways were discussed in details.

**Conclusion:**

Pathway analysis indicates that the introduced central genes of the network can be considered as biomarkers of gastric atrophy.

## Introduction

 Gastric atrophy is characterized with progressive decrease of glandular structures accompanied with mucosa changes to atrophic (1). Gastric atrophy is recorded as “loss of glands” in stomach. This process may follow ulceration accompanied with damage of glandular layer. In another case which occurs more frequently; it results from a prolonged inflammation. In additional definition atrophy is considered as “loss of specialized cells” (2). There are evidences that gastric atrophy can be considered as risk factor of cancer. It is an important state which causes pathologically gastric cancer. Gastric atrophy diagnosis is improved by endoscopy (especially by new criteria of this method) (3). Variety of studies revealed molecular aspects of gastric atrophy. The role of different genes such as interleukin-2, cyclooxygenase-2 and several genes in the gastric atrophy especially in Helicobacer pylori inducing disease, is investigated and highlighted (4, 5). Helicobacter pylori infection is mentioned as one of important causes of gastric atrophy (6, 7).

Different diseases are assessed via PPI network analysis to discover new molecular aspect of disorder (8-11). In this approach all reported genes or other molecular reagents such as proteins or metabolites which are correlated to the disease are collected and organized in an interactome which is an integrated and interacted unit (12, 13). Based on diverse roles which the elements play in PPI network, they rank and screen (14). Variety of factors are introduced for screening which central parameters such as degree and betweenness are the important ones (15). In the other hand GO analysis is a useful tool to find terms such as biological processes, molecular function, cellular component, and biochemical pathways related to the query genes (16). The identified terms can be clustered by using statistical parameters (17). In this tactic new molecular prospective of disorder will be represented (18). In the present study the genes related to the gastric atrophy are retrieved from STRING database and organized in the PPI network. The network analyzed and the central genes determined and enriched to determine the related biochemical pathways. The pathways discussed to present new views of gastric atrophy. 

## Methods

The recorded genes in STRING database up dated at 2017(19) related to gastric atrophy from its disease query were retrieved. The genes interacted by Cytoscape software version 3.6.0 (20) to construct an interactome. The PPI network was analyzed by network analyzer a plug-in of Cytoscape and the topological properties of the network were assessed. Two important central parameters (degree and betweenness centrality) were considered for screening of the nodes. The top nodes based on degree (degree more than mean + 2SD) were selected as hub-nodes and the nodes characterized as top 5% based on betweenness were determined as bottleneck-nodes (12, 17). The common hub and bottleneck nodes were identified as hub-bottleneck nodes. Cluster ONE (21, 22) application of Cytoscape was used to determined significant clusters of the network and P-value less than 5% was considered. The elements of the main cluster enriched via gene ontology by ClueGO (v. 2.5.0) plug-in of Cytoscape (23, 24). Related biochemical pathways to the nodes of main cluster were determined; at least three genes per term and three percent attribution in the term were considered. The terms were grouped based on kappa score (25). For better discussion restricted condition including at least seven genes per term and five percent attribution in the term were considered. The terms were discussed in details. 

## Results

**Figure 1:**
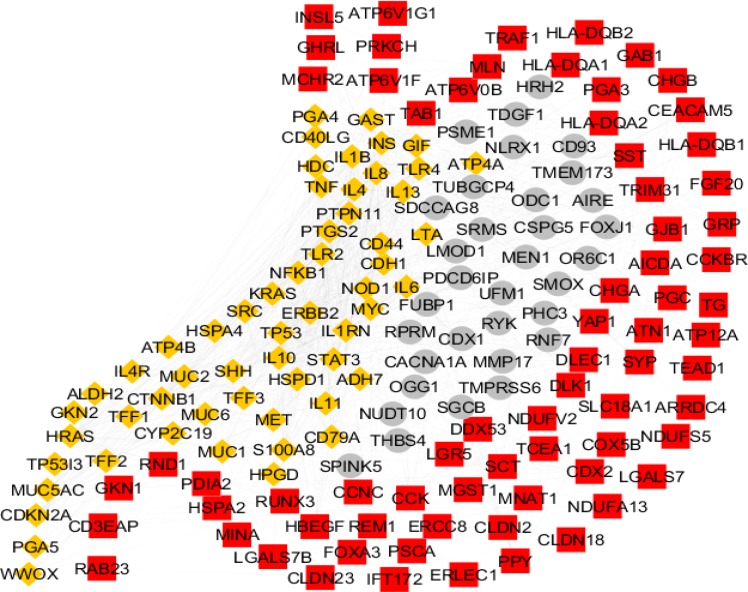
The number of 206 genes related to gastric athrophy were organized in the PPI network. The network was included a main connected component containing 161 nodes and 45 isolated nodes. Confidence score was 0.4. The main connected component was included cluster-1, 2, and 3 which are colored in red, yellow, and grey respectively

There are 206 genes related to gastric atrophy. As it is shown in the figure 1 the numbers of 161 genes among them are included in the PPI network and the others (45 genes) were isolated. The genes are organized in three clusters including cluster-1 (the red color nodes), cluster-2 (the yellow colored genes), and cluster-3 (the grey colored nodes). The clusters 1-3 contains 71, 57, and 33 nodes, respectively. The network was analyzed and the hub and bottleneck nodes were determined. The common hub and bottleneck nodes including INS, TP53, IL6, TNF, SRC, MYC, and IL8 were identified as hub-bottleneck nodes (see table 1). Degree distribution was fitted in a power law line with y=axb which a and b are equal to 25.745 and -0.840, respectively. Correlation and R-squared (computed on logarithmized values) are 0.922 and 0.749, respectively. Therefore, the network is a scale free network.

**Table 1:** Ten hub-nodes of the analyzed network are presented. There are eight bottleneck nodes which seven of them are hub-nodes (see the bold hub-nodes) and another one is GAST. BC, D, and DS are betweenness centrality, degree, and disease score respectively

R	name	description	BC	D	DS
1	INS	insulin	0.15	62	0.67
2	TP53	tumor protein p53	0.18	59	1.57
3	IL6	interleukin 6 (interferon, beta 2)	0.04	51	0.59
4	TNF	tumor necrosis factor	0.05	48	1.11
5	SRC	v-src sarcoma (Schmidt-Ruppin A-2) viral oncogene homolog (avian)	0.09	48	0.98
6	MYC	v-myc myelocytomatosis viral oncogene homolog (avian)	0.06	47	0.85
7	IL8	interleukin 8	0.05	45	1.58
8	PTGS2	prostaglandin-endoperoxide synthase 2 (prostaglandin G/H synthase and cyclooxygenase)	0.04	43	1.29
9	HRAS	v-Ha-ras Harvey rat sarcoma viral oncogene homolog	0.02	39	0.56
10	NFKB1	nuclear factor of kappa light polypeptide gene enhancer in B-cells 1	0.02	38	0.63

**Table 2:** The number of 18 terms among 31 resulted terms from enrichment of elements of cluster-2 which are organized in the eight groups are presented. P-value≤0.05, 7≤genes/term, and 5≤%genes/term. The underlined number refers to repeat of term. The underlined number corresponding to repeat of the term

R	Terms	Genes/Term	%Genes/Term
1	Kaposis sarcoma associated herpesvirus infection	11	6
2	AGE-RAGE signaling pathway in diabetic complication	8	8
3	Toxoplasmosis	7	6
4	Hepatitis C	7	5
5	Cellular senescence	8	5
6	Hepatitis B	12	8
7	Measles	9	7
8	Hepatitis B_1	12	8
9	Proteoglycans in cancer	15	7
10	Gastric cancer	10	7
11	Cellular senescence_1	8	5
12	Kaposis sarcoma associated herpesvirus infection_1	11	6
13	Chornic myeliod leukemia	7	9
14	Tuberculosis	9	6
15	Herpes simplex infection	8	5
16	Cytokin-cytokin receptor infection	12	5
17	Hematopoietic cell lineage	7	8
18	T cell receptor signaling pathway	7	8
19	Leishmaniasis	8	12
20	Malaria	9	19
21	Inflammatory boweldisease (IBD)	11	18
22	Hematopoietic cell lineage_1	7	8
23	AGE-RAGE signaling pathway in diabetic complication_1	8	9
24	Measles_1	9	8
25	Herpes simplex infection_1	8	6
26	Inflammatory boweldisease (IBD)_1	11	18

**Figure 2:**
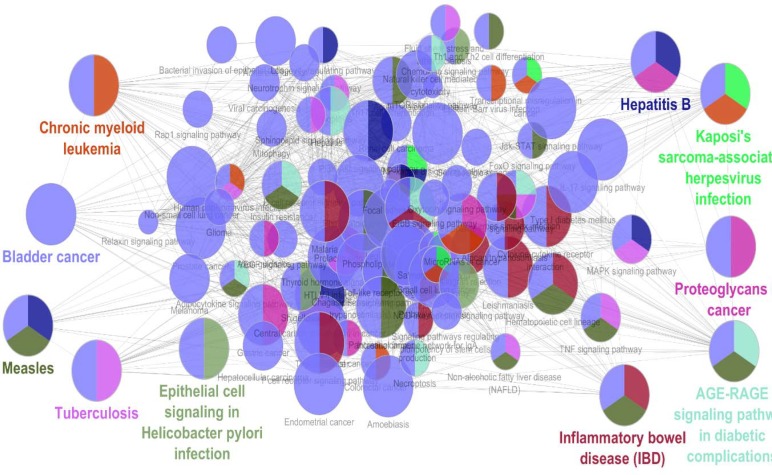
The elements of cluster-2 are enriched by ClueGO v. 2.5.0. The biochemical pathways are extracted from KEGG_20.11.2017: 7282. The number of 71 terms are organized in the 10 groups based on kappa score. Presence of at least three genes/term and 3% genes/term were considered. The groups name is shown in different colors

**Table 3:** The number of 13 terms among 31 resulted terms from enrichment of elements of cluster-2 which are organized in one group are presented. P-value≤0.05, 7≤genes/term, and 5≤%genes/term. The underlined number refers to repeat of term. The repeated terms are shown with underlined numbers

R	Terms	Genes/Term	%Genes/Term
1	NF-Kappa B signaling pathway	8	8
2	Cellular senescence_2	8	5
3	Toll-like receptor signaling pathway	7	7
4	Jak-STAT signaling pathway	10	6
5	Hematopoietic cell lineage_2	7	7
6	IL-17 signaling pathway	10	11
7	T cell receptor signaling pathway_1	7	7
8	AGE-RAGE signaling pathway in diabetic complication_2	8	8
9	Pertussis	8	11
10	Legionellosis	8	14
11	Leishmaniasis_1	8	11
12	Chagas disease (American trypanosomiasis)	8	8
13	Malaria_1	9	17
14	Toxoplasmosis_1	7	6
15	Amoebiasis	9	9
16	Tuberculosis_1	9	5
17	Hepatitis C_1	7	5
18	Hepatitis B_2	12	8
19	Measles_2	9	7
20	Kaposis sarcoma associated herpes virus infection_2	11	6
21	Proteoglycans in cancer_1	15	7
22	Endometrial cancer	7	12
23	Prostate cancer	7	7
24	Bladder cancer	9	22
25	Chronic myeloid leukemia	7	9
26	Gastric cancer_1	10	6
27	Inflammatory bowel disease (IBD)_2	11	17
28	Rheumatoid arthritis	7	8

Due to presence of all hub-bottleneck nodes in the cluster-2, the elements of cluster-2 were enriched by ClueGO v. 2.5.0. The related chemical pathways were extracted via KEGG and are presented in the figure 2. More restrictions (presence of at least seven genes in term and 5% attribution of genes in the term) were considered and the finding were tabulated in the tables 2 and 3. The number of 31 terms were organized in nine groups including: Kaposis sarcoma associated herpesvirus infection, AGE-RAGE signaling pathway in diabetic complication, Hepatitis B, Proteoglycans in cancer, Chornic myeliod leukemia, Tuberculosis, Herpes simplex infection, Inflammatory bowel disease (IBD), Measles, and bladder cancer.

## Discussion

As it is shown in the figure 1 the numbers of 161 genes are organized in a scale free network. It is possible that several genes among them play critical roles in the network. Presence of limited numbers of crucial genes in network (the seven hub-bottleneck nodes which are shown in table 1) is corresponded to successful screening of the genes by network analysis. Surprisingly, all of the critical nodes are presented in the cluster-2. Since this cluster includes 57 nodes, it seems these genes play central role in the network. There is evidence that the elements of a cluster are related to each other and manage similar functions. Based on this object the mentioned 57 genes were enriched and the biochemical pathways were identified. The results of enrichment and more restricted analysis are shown in the figure 2 and tables 2 and 3, respectively. The 31 terms which organized in the nine groups were introduced. Previous studies indicate that understanding of the related biochemical pathways is useful to know mechanism of disorder. In the following part, the roles of these highlighted groups in gastric atrophy are discussed. 

The biggest group which includes 28 terms is represented as bladder cancer cluster. This collection contains 9o% of the identified total terms. It is reported that in the case of gastritis due to *Helicobacter pylori *infection NF-κB activity increases in the epithelial cells (26). Based on literature; inhibition of FoxM1 hints to cellular Senescence. FoxM1 expression increases in gastric cancer (27). The role of Toll-like receptors in gastric cancer and gastric atrophy is discussed in details (28). Assessment of role of inflammatory factor IL-17 by T helper cells and Jak-STAT signaling pathway in gastritis indicates that these two agents are related to gastric tumourigenesis (29). Ohnishi *et al.* reported occurrence of gastrointestinal and hematopoietic neoplasms after infection with *Helicobacter pylori* (30). It is reported that after gastric ulcer healing RAGE mRNA expression is increased (31). As it is presented in the figure 2, this pathway is related to the bladder cancer and measles. Role of *Helicobacter pylori* in inflammatory bowel disease is confirmed (32). In the other hand, this microorganism is the main mediator of gastric atrophy. A relationship between *Bordetella pertussis *toxin secretion protein and *Helicobacter pylori* secretion is found (33). There is evidence that sever malaria infection leads to increased gastroduodenal permeability band hepatitis C virus infection is detected and discussed (34). As it is reported, glypcian-3 which is a tumor suppressor gene belongs to proteoglycan family. Down-regulation of this gene in gastric cancer is described (35, 36). Frequent connections between the introduced pathways in figure 2, indicate that all of them are related directly or indirectly to the gastric atrophy. If this statement be accepted, it can be concluded that cluster-2 is a right core of the main genes which are involved in the disease. As it is mentioned in the result section, this cluster contains the seven central genes. Therefore it is a logical conclusion that the central genes are a suitable biomarker panel for gastric atrophy. Presence of IL-6 and IL-8 as two inflammatory reagents beside TP53 and TNF as two well-known tumor indicators in the introduced biomarker panel, are corresponding to the complex nature of gastric atrophy.

Biochemical pathway analysis of gastric atrophy represents a new prospective of molecular mechanism of disorder. The related biomarkers were proposed which are corresponded to the roles of inflammation and cancerous pathways in this disease. Based on the findings more investigation can lead to introduce safe diagnostic method for the disease instead of endoscopy.
